# Online Patient Education Materials on Iron Deficiency Anemia Are Too Difficult to Read and Low Quality: A Readability and Quality Analysis

**DOI:** 10.7759/cureus.46902

**Published:** 2023-10-12

**Authors:** Sajal Kulhari, Aaron B Ahn, James Xu, Jasmine Rhee, Gregory Cooper

**Affiliations:** 1 Gastroenterology, Case Western Reserve University School of Medicine, Cleveland, USA; 2 Dermatology, Case Western Reserve University School of Medicine, Cleveland, USA; 3 Nursing, Emory University Nell Hodgson Woodruff School of Nursing, Atlanta, USA; 4 Gastroenterology, University Hospitals Cleveland Medical Center, Cleveland, USA

**Keywords:** iron deficiency anemia (ida), preventative medicine, online health information, anemia, quality improvement, readability, patient education

## Abstract

Introduction

Patients increasingly rely on online health information to understand and manage their diseases. Concerns about the quality and readability of these materials have been reported in the literature. Poor quality and difficult-to-read information lead to delayed diagnoses and adverse outcomes. We assessed the quality and readability of online health information about iron deficiency anemia (IDA) on Google.

Method

We searched for online web pages using the term "iron deficiency anemia"on Google. One hundred and twelve out of 200 web pages were included. We assessed web page typology, readability, the Journal of the American Medical Association (JAMA) score, the DISCERN score, and the Health on the Net Foundation certification (HONcode). Statistical analysis was performed with R version 4.2.2.

Result

Non-profit and scientific journal web pages were the most common. Scientific journal web pages were of the highest quality. News web pages were the most readable. The first Google Page web pages did not have greater JAMA scores or lower Flesch-Kinkaid Reading Grade Level (FKGL) and Simple Measure of Gobbledygook (SMOG) scores. Forty-six percent of all web pages were high-quality. Web pages on the first Google page were more likely to have HONCode certification.

Conclusion

We highlight gaps in the readability and quality of online information about IDA. Online web pages exceeded the recommended reading level for patients. Most web pages were low quality; only a quarter were HONcode-certified; and the first Google page web pages were not higher in quality than the later web pages on search.

## Introduction

Iron deficiency anemia (IDA) accounts for 30%-50% of all cases of anemia [1]. Certain cases of IDA are more alarming than others [2]. For example, IDA within the context of the elderly is especially concerning for colorectal cancer [3]. On the other hand, IDA in the context of pregnancy is more often a consequence of the increased metabolic demand of pregnancy or dilution than a pathologic process [4]. The several causes of IDA and their varying severity present a notable challenge as patients navigate health information.

Increasingly, patients turn to online health information to fortify their understanding of their condition and take an active role in their disease management [5,6]. Upwards of half of all patients have reported utilizing the internet to gather more information about their health [7]. This highlights important concerns regarding the quality and readability of these materials [8]. Health information on topics such as IDA is complex and requires high quality in order to prevent the spread of misinformation or misleading information. Furthermore, limitations in the readability of online materials disproportionately affect patients who have low health literacy. Thirty-six percent of all Americans are at a basic or below-basic level of health literacy [9], with the average American reading at an eighth-grade level [10]. While the American Medical Association (AMA) recommends patient health information be written at the sixth-grade level [11], previous studies have shown the reading level of online health information to be considerably higher than this standard [12].

The consequences of low-quality and inadequately readable health information are serious. Previous studies have reported that poor readability of written information can prolong recovery, delay hospitalizations, and increase the rate of complex adverse outcomes [13]. Low-quality health information can also be misleading, resulting in delayed or missed diagnoses [14]. It is critical to assess the quality and readability of online health information, and increasingly, topics across medicine have been studied with this very aim.

Our study aims to assess the quality and readability of online health information within the context of IDA. Furthermore, we assess the types of websites with the highest quality, the differences in quality and readability between the first Google web page and later pages, and the reading level of web pages of high and low quality.

## Materials and methods

Data collection

We searched for online web pages using the term "iron deficiency anemia" on Google (Google LLC, Mountainview, CA). We chose a simple and neutral term to yield a large sample of websites and to mirror a hypothetical patient who would be searching for information on IDA. Furthermore, Google is the most frequently used search engine by patients [15]. Cookies and browser history were cleared prior to the search. The first 200 web pages of our search result were recorded, as is consistent with previous studies [16]. Web pages were then excluded based on the following criteria: web pages contained no information about IDA; web pages were in a language other than English; web pages required subscription or registration; web pages were greater than three clicks from providing information; web pages repeated or sourced information from a previously assessed site; and web pages that hosted primarily video content.

Stratifying web pages by type

Our study divided web pages into eight typologies based on similar studies within the literature [17]. These include commercial, government, health portals, news, non-profit, professional, scientific journals, and others. Web page type was discerned by two medical students at a Midwestern metropolitan medical institution working independently (SK, AA). Commercial web pages aim to sell products or services. Government refers to web pages that are operated by a government agency. Health portals refer to websites that provide health information on a variety of different health topics. News refers to web pages operated by news media. Non-profit refers to web pages that are hosted on an education or charity-focused website and are not profit-oriented. Professional refers to web pages created by an agency or person with professional qualifications (i.e., a doctor of medicine (MD) or authorized medical attendant (AMA)). Scientific journals refer to web pages that are academic and peer-reviewed. If web pages did not fit one of these typologies, they were labeled “other”. Inconsistencies in typology were resolved by the researchers coming to an agreement. Cohen’s kappa coefficient of agreement between the included studies was 0.85 [18, 19].

Quality assessments

The quality of health information was assessed using three metrics commonly used in similar studies: the Journal of American Medicine Association (JAMA) score, the DISCERN score, and the Health on the Net Foundation certification (HONcode) [17].

The JAMA scores assess four factors: authorship, attributions, disclosure, and currency [8]. Specifically, authorship refers to the presence of all authors and contributors and their affiliations and qualifications. Attribution refers to all sources and references being clearly presented. The disclosure pertains to a clear demonstration of website ownership; conflicts of interest and funding sources should also be transparent. Lastly, currency refers to the presence of dates when content was first posted and updated.

Meeting each criterion confers one point; the absence of a criterion confers 0 points. Therefore, four points is the maximum score a web page can attain, and 0 is the minimum. Web pages with three to four points were considered high-quality [16]. Two researchers (SK and AA) scored the included web pages for JAMA scores, and inconsistencies were resolved by the researchers coming to an agreement. The kappa score of the JAMA scores collected by two independent researchers was 0.70.

The DISCERN questionnaire is brief and allows authors to reliably assess the quality of written consumer health information [20]. The DISCERN questionnaire consists of 16 questions, rated on a five-point scale ranging from no to yes. For example, a DISCERN score of five is given if the answer to the question is a definite ‘yes’, one is given if the answer to the question is a definite 'no', and scores of two to four represent answers that partially meet criteria. The questionnaire is split into three separate sections. Section one (questions one to eight) relates to general information about the websites, such as the reliability of the publication and the trustworthiness of the source authors used. Section two (questions nine to 15) focuses on specific details regarding the treatment websites. Section three (question 16) is the overall quality rating based on the individual’s judgment of the quality of the source. The points for DISCERN range from 16 to 80. The scores are evaluated as 63-80 points (excellent), 51-62 points (good), 39-50 points (average), 28-38 points (poor), and 16-27 points (very poor) [21]. Scores from two independent reviewers were averaged to get the mean DISCERN score.

The HONcode certification ensures the quality of information on a website and is provided by an independent organization. Certification ensures assessments of quality, confidentiality, neutrality, trust, support, and community [22]. Researchers in our study assessed the presence of HONcode certification on the web pages of our sample.

Readability assessment

We utilized the Flesh-Kincaid grade level (FKGL) and the Simple Measure of Gobbledygook (SMOG) to measure the readability of all web pages in our sample. The FKGL is determined by a formula that considers the total number of words per sentence and the total number of syllables per word [23]. For example, a FKGL level of 8.4 indicates the material is written at an 8th to 9th-grade reading level. The SMOG score is determined by a formula that considers the total polysyllables and total sentences used for a text with at least 30 sentences [24]. In both assessments, higher scores indicate a more difficult reading level.

Ethics statement

In this study, the researchers did not assess any human participants or animals. Websites that anyone can access were assessed. Therefore, there was no need for the approval of the ethics committee for this study.

Statistical analysis

The statistical analysis of the data was performed using R version 4.2.2 (The R Core Team, R Foundation for Statistical Computing, Vienna, Austria). The Shapiro-Wilk test was used to evaluate the normality of the distribution. Mann-Whitney tests and Student's t-tests were executed for comparisons of continuous variables (means) between two groups and Kruskal-Wallis one-way ANOVA/one-way ANOVA tests between more than two groups. For categorical variables, comparisons of groups were performed using Chi-square tests. Statistical significance was set at p < 0.05.

## Results

Overview

Of 200 articles from our initial search, 88 were excluded due to them requiring a subscription, having no information about IDA, being in a language other than English, having duplicates, and requiring more than three clicks to access relevant information. After evaluation of the remaining websites (n=112), we found the overall mean scores for the readability indices FKGL and SMOG were 8.77±2.49 (95% CI: 8.31-9.23) and 7.69±2.05 (95% CI: 7.31-8.07) (Table 1).

**Table 1:** Readability and quality of all included web pages FKGL: Flesch-Kinkaid Reading Grade Level; SMOG: Simple Measure of Gobbledygook; JAMA: Journal of the American Medical Association; HONcode: Health on the Net Foundation certification; avg: average; stdev: standard deviation

Total web pages	FKGL_avg	FKGL_stdev	SMOG_avg	SMOG_stdev	JAMA_avg	JAMA_stdev	DISCERN_avg	DISCERN_stdev	HONcode certified_percentage
n=112	8.770535714	2.489568811	7.69375	2.049270635	2.5625	1.072012925	3.514285714	0.8330836177	0.2589285714

The total mean was 3.51 ± 0.83 for DISCERN and 2.56 ± 1.07 for JAMA. Of 112 websites, we classified 51 (46%) as high-quality (JAMA score ≥ 3) (Table 2).

**Table 2:** Differences in readability and quality between high-quality pages and low-quality pages FKGL: Flesch-Kinkaid Reading Grade Level; SMOG: Simple Measure of Gobbledygook; JAMA: Journal of the American Medical Association; HONcode: Health on the Net Foundation certification; avg: average; stdev: standard deviation

Quality	FKGL_avg	FKGL_stdev	p-value (t-test)	SMOG_avg	SMOG_stdev	p-value (t-test)	JAMA_avg	JAMA_stdev	p-value (t-test)	DISCERN_avg	DISCERN_stdev	p-value (t-test)	HONcode certified_percentage	p-value (chi-square)
Low Quality (n=61)	7.71639344	1.31759634	2.16E-06	6.93114754	0.95682478	4.59E-05	1.68852459	0.46693981	2.20E-16	3.00983607	0.65388707	8.16E-16	0.24590164	0.8984
High Quality (n=51)	10.0313725	2.94940606		8.60588235	2.58189169		3.60784314	0.4930895		4.11764706	0.58811764		0.2745098	

Of all websites, 25.9% (29) were HONcode-certified (Table 3).

**Table 3:** Differences in readability and quality between HONcode-certified pages and non-HONcode-certified pages FKGL: Flesch-Kinkaid Reading Grade Level; SMOG: Simple Measure of Gobbledygook; JAMA: Journal of the American Medical Association; HONcode: Health on the Net Foundation certification; avg: average; stdev: standard deviation

HONcode	FKGL_avg	FKGL_stdev	p-value (t-test)	SMOG_avg	SMOG_stdev	p-value (t-test)	JAMA_avg	JAMA_stdev	p-value (t-test)	DISCERN_avg	DISCERN_stdev	p-value (t-test)	HONcode certified_percentage	p-value (Chi-square)
Not certified (n=83)	8.92289157	2.60231254	0.2304	7.7626506	2.17848184	0.4947	2.4939759	1.06351344	0.2632	3.25542169	0.75227347	3.19E-10	0	NA
Certified (n=29)	8.33448276	2.11479075		7.49655172	1.64175054		2.75862069	1.09071315		4.25517241	0.5717056		1	

Typology

**Figure 1:**
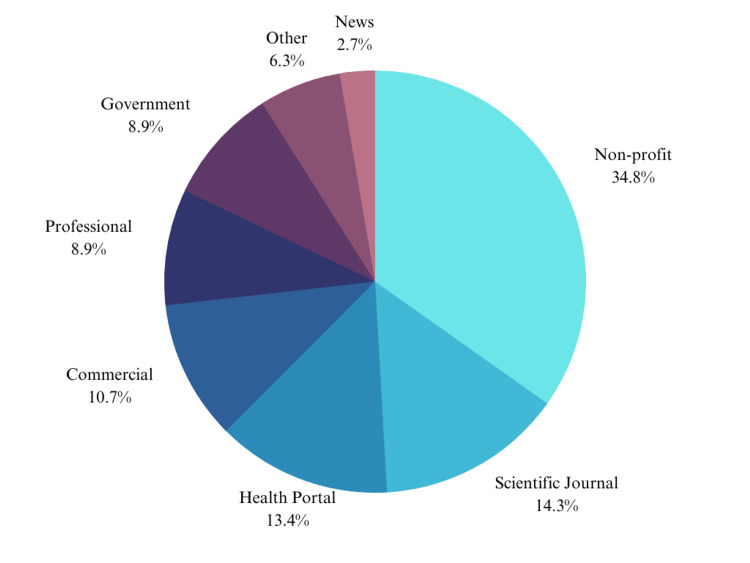
Distribution of web page typology

From the 112 websites, the typologies were non-profit (34.8%), scientific journal (14.3%), health portal (13.4%), commercial (10.7%), professional (8.9%), government (8.9%), other (6.3%), and news (2.7%) (Figure 1).

Evaluating typologies by quality, we found 100% of scientific journals, 66.6% of news sites, 60% of government sites, 50% of professional sites, 46.7% of health portals, 33.3% of non-profit sites, 16.7% of commercial sites, and 0% of “other” websites were high-quality websites (Table 4).

**Table 4:** Differences in readability and quality by type of web page FKGL: Flesch-Kinkaid Reading Grade Level; SMOG: Simple Measure of Gobbledygook; JAMA: Journal of the American Medical Association; HONcode: Health on the Net Foundation certification; avg: average; stdev: standard deviation

Type	FKGL_avg	FKGL_stdev	p-value (ANOVA)	SMOG_avg	SMOG_stdev	p-value (ANOVA)	JAMA_avg	JAMA_stdev	p-value (ANOVA)	DISCERN_avg	DISCERN_stdev	p-value (ANOVA)	HONcode certified_percentage	p-value (Chi-square)
Commercial (n=12)	8.85	1.655569114	2.72E-07	7.316666667	0.9320879137	1.17E-06	1.916666667	0.9003366374	2.86E-06	3.1	0.7032392584	1.39E-05	0.25	0.02305
Government (n=10)	8.95	2.68131394		7.87	2.418470591		2.8	0.7888106377		3.92	0.543241301		0.3	
Health portal (n=15)	8.246666667	2.319441641		7.426666667	1.995160812		2.533333333	0.9904304019		3.68	0.7839460623		0.6	
News (n=3)	7.3	0.6		7	0.1		3.333333333	1.154700538		3.766666667	1.205542755		0.3333333333	
Non-profit (n=39)	7.92051282	1.438050487		6.876923077	1.031978564		2.358974359	1.063439788		3.287179487	0.8414209433		0.1282051282	
Other (n=7)	7.471428571	0.5908025212		6.985714286	0.5814595756		1.428571429	0.5345224838		2.514285714	0.4775931722		0	
Professional (n=10)	8.59	2.401133991		8.04	1.429218901		2.7	1.059349905		3.79	0.5342700108		0.4	
Scientific journal (n=16)	12.11875	3.202336387		10.33125	3.063270311		3.6875	0.4787135539		4.1875	0.5920304046		0.25	

The most readable to least readable typologies using FKGL ranged from news, other, non-profit, health portal, professional, commercial, government, and scientific journals.

Web pages on the first Google page

Of 112 websites, seven were found on the first page (Table 5).

**Table 5:** Differences in readability and quality between the first Google page and later Google pages FKGL: Flesch-Kinkaid Reading Grade Level; SMOG: Simple Measure of Gobbledygook; JAMA: Journal of the American Medical Association; HONcode: Health on the Net Foundation certification; avg: average; stdev: standard deviation

Google page	FKGL_avg	FKGL_stdev	p-value (t-test)	SMOG_avg	SMOG_stdev	p-value (t-test)	JAMA_avg	JAMA_stdev	p-value (t-test)	DISCERN_avg	DISCERN_stdev	p-value (t-test)	HONcode certified_percentage	p-value (Chi-square)
Later Google pages (n=105)	8.77058824	2.56254778	0.9992	7.68921569	2.11458429	0.9122	2.52941176	1.05027938	0.3978	3.43431373	0.80965646	0.001202	0.21568627	0.003093
First Google page (n=7)	8.77	1.65801086		7.74	1.26947058		2.9	1.28668394		4.33	0.6254776		0.7	

The DISCERN scores of the web pages on the first Google page were significantly greater than web pages on later pages (4.33 vs. 3.43; (p =0.001)). However, no significant differences in JAMA scores (p = 0.40), FKGL (p=0.99), or SMOG (p=0.91) between the websites on the first page of Google and the remaining websites were found. Web pages on the first page were significantly more likely to have HONCode certification (p=0.003). The likelihood of different types of web pages being HONcode-certified is the following: health portals (60%), professional (40%), news (33.3%), government (30%), scientific journals (25%), commercial (25%), non-profit (12.8%), and others (0%).

High-quality vs. low-quality web pages

Of 112 websites, we classified 51 (46%) as high-quality (JAMA score ≥ 3) (Table 2). We found a significant difference in FKGL and SMOG scores between high- and low-quality web pages. On average, FKGL and SMOG scores were significantly higher on high-quality web pages compared to low-quality web pages (FKGL: 10.03 vs. 7.72 (p=0.000002); SMOG: 8.61 vs. 6.93 (p = 0.00005)). We also found a significant difference in DISCERN scores between high and low-quality web pages, with high-quality web pages having higher mean DISCERN scores (4.12 vs. 3.01; (p=8.16x10-16).

Outcomes By HONcode

Of 112 websites, 29 (26%) web pages were HONcode-certified (Table 3). There was no significant difference reported between FKGL (p=0.23) and SMOG (p=0.49) scores comparing HONcode versus non-HONcode web pages. Similarly, no significant difference (p=0.26) was found when comparing JAMA scores. DISCERN scores were significantly different between HONcode web pages versus non-HONcode web pages, with DISCERN scores higher for HONcode web pages (4.26 vs. 3.26; (p=3.19x10-10).

## Discussion

The quality of online health information is critical to promoting access and health literacy among patients. Previous studies have documented the lack of quality of online health information and its consequences, such as misinformation, misleading information, and delayed diagnosis [25-27]. While standardized guidelines have been created for online health information, such as JAMA and HONCode [8,22], considerable variability exists in the quality of online health information. Our study sought to assess the readability and quality of online health information on iron deficiency anemia (IDA), with the following questions driving our analysis: Do online information sources provide high-quality information? What is the distribution of websites of different types in providing information on IDA? Which website typologies have greater quality and readability? What is the readability of online information on IDA? How do readability and quality compare between the websites on the first page and the websites on later pages? How does readability compare between high-quality and lower-quality websites?

The most readable type of web page was news, and the least readable type was a scientific journal. Similar to previous studies [2,15,16], we found an inverse relationship between readability level and quality, with high-quality articles being less readable on average.

We hypothesized that the first Google page web pages would have greater readability and quality in comparison to later pages due to their higher ranking by the Google algorithm. However, we found mixed results. No differences were found in readability between web pages on the first page and later pages. With regards to quality, no significant differences in JAMA scores were found between websites on the first page of Google versus the remaining websites, a finding validated by previous studies [15,28]. When employing DISCERN instead of JAMA, however, first-page results were of higher quality than later web pages. This may be explained by the difference in the way JAMA and DISCERN measure quality; while JAMA focuses on authorship, attribution, disclosure, and currency [8], DISCERN offers a more subjective and detailed method for assessing quality [20].

Since more than 25% of people click on the very first search result and the majority click on articles within the first Google page [29], we conclude that the vast majority of patients read articles on IDA that are far above their reading level. It remains unclear if patients are obtaining higher-quality health information on the first Google page than on later pages. Our analysis also questions whether the Google search algorithm accounts for readability when forming web page rankings.

The most frequent typologies were non-profit, scientific journal, health portal, and commercial. Scientific journals had the highest quality typology and all scientific journal web pages were high quality. Kaya & Görmez [17] and Lim et al. [30] similarly found scientific journals to have the highest quality typology. In contrast, Basavakumar et al. [15] and Chumber et al. [28] found non-profit and health portal web pages to be of the highest quality, respectively. In our study, less than one in five commercial web pages were of high quality. Similarly, previous studies have found commercial web pages to be among the lowest-quality sources of online health information [16,17,28]. Commercial web pages’ low quality and profit-driven incentives pose a threat to the health literacy of patients.

One in four web pages was HONcode-certified. First-page web pages and higher-quality web pages were more likely to be HONcode-certified. The typologies most likely to have HONcode certification were Health Portal and Professional. The typologies least likely to have HONcode certification were other and commercial. Interestingly, we found no significant difference in readability scores or JAMA scores between HONcode-certified web pages and non-certified web pages. We also found no difference in the likelihood of HONcode certification between high-quality and low-quality web pages. Furthermore, the likelihood of scientific journal web pages being HONcode-certified was only 25%, despite significantly higher JAMA and DISCERN scores than other typologies. It is therefore inappropriate to assume web pages are harder to read or are of lower quality simply because they lack HONcode certification.

Perhaps the most compelling explanation for this finding is that HONcode certification is not easily accessible. HONcode certification requires a fiscal contribution according to the type of website and its visibility: a commercial website with high visibility costs $385 versus a non-commercial website with low visibility, which costs $65. Further, the contribution must be renewed annually when the site is reevaluated [30].

Our study has many limitations. We only evaluated websites that were written in English. Further, we only used one search engine, Google, and one search term, ‘iron deficiency anemia', to discover relevant web pages. This is because past studies have shown that the majority of consumers of online health information utilize Google searches. Future studies could expand the scope of this study by including web pages in non-English languages and search engines other than Google. Videos and other resources involving sound (i.e., Khan Academy, YouTube) could not be analyzed for readability scores and were excluded. Due to variability in Google rankings by consumer, patients may see web pages in a different order than in our study. To minimize this effect, we cleared cookies and browser history prior to searching. Lastly, applying the JAMA and DISCERN scores required subjective observations, which may vary between the evaluators in the study. Nevertheless, the Cohen's Kappa coefficient score between the evaluators was 0.70, indicating strong agreement.

## Conclusions

We report the overall readability of online web pages on IDA as exceeding the recommended reading level for patients. Furthermore, the majority of the web pages we reviewed were of low quality; only a quarter were HONcode-certified, and the prevalence of high-quality web pages on the first Google page was no different than that of later pages. Our findings emphasize the gaps in readability and quality of online information about IDA and demonstrate the importance of incorporating readability and quality considerations for online health information. A particularly significant finding of our paper is the lack of consideration of readability and quality within Google rankings. The first page of Google results represents the web pages most encountered and used by patients, and these should therefore be of the highest quality and readability in comparison to later web pages. We also report the concerning inverse relationship between health information quality and readability, with the highest-quality web pages having the lowest readability. Ultimately, patients should be able to easily access and understand health information on the internet and should be able to rely on the quality of this information just as much as they do on their providers.
